# Previously Unrecognized Tracheobronchopathia Osteochondroplastica Diagnosed Following Unexpected Difficult Tracheal Intubation During Robotic Surgery: A Case Report and Literature Overview

**DOI:** 10.1002/ccr3.73504

**Published:** 2026-09-23

**Authors:** Ottavio Paolo Volino, Loreto Maria, Palma Antonella, De Simone Anna, Tomasello Antonio, Mainini Marco, Pisanti Massimo, Villani Romolo

**Affiliations:** ^1^ Department of Anesthesia Cardarelli Hospital Naples Campania Italy; ^2^ Department of Women's, Children's, General and Specialized Surgery Università degli Studi della Campania “L. Vanvitelli” Naples Campania Italy

**Keywords:** airway management, difficult tracheal intubation, rigid bronchoscopy, tracheal stenosis, tracheobronchopathia osteochondroplastica, videolaryngoscopy

## Abstract

Previously unrecognized tracheobronchopathia osteochondroplastica caused unexpected failure of endotracheal tube advancement despite optimal videolaryngoscopic visualization during robotic surgery. Bronchoscopy established the diagnosis and enabled definitive treatment with rigid bronchoscopy. This case highlights the importance of suspecting occult tracheal pathology and avoiding repeated traumatic intubation attempts.

## Key Clinical Messages

Failure to advance an endotracheal tube despite optimal videolaryngoscopy should raise suspicion of occult tracheal pathology. Early bronchoscopy and multidisciplinary management are essential for safe airway control and definitive treatment.

## 1 Introduction

Tracheobronchopathia osteochondroplastica (TPO), also referred to as tracheopathia osteochondroplastica, is a rare benign disorder characterized by multiple submucosal osteocartilaginous nodules arising from the cartilaginous rings of the trachea and main bronchi, with typical sparing of the posterior membranous wall [1, 2]. Although its pathogenesis remains poorly understood, TPO generally follows an indolent clinical course and is often asymptomatic or associated with nonspecific respiratory symptoms. Consequently, it is frequently diagnosed incidentally during bronchoscopy or cross‐sectional imaging performed for unrelated indications [3].

Clinical manifestations include chronic cough, dyspnea, recurrent respiratory tract infections, hemoptysis, and, in advanced cases, clinically significant airway obstruction [4]. In anesthetic practice, TPO represents an uncommon but potentially critical cause of unexpected difficult tracheal intubation, particularly when osteocartilaginous lesions involve the subglottic region and proximal trachea [5].

Conventional preoperative airway assessment primarily estimates the likelihood of difficult laryngoscopy and provides limited information about distal airway abnormalities [6]. Therefore, patients with clinically silent tracheal disease may have an apparently normal airway examination despite severe subglottic or tracheal stenosis. Failure to advance an endotracheal tube despite optimal laryngeal exposure should prompt consideration of occult subglottic or tracheal pathology, including post‐intubation tracheal stenosis, tracheomalacia, tracheal neoplasms, and tracheobronchopathia osteochondroplastica [5, 7].

We describe an elderly woman undergoing robot‐assisted hysterectomy in whom previously unrecognized tracheobronchopathia osteochondroplastica was diagnosed after unexpected resistance to endotracheal tube advancement despite excellent videolaryngoscopic visualization. This case highlights the discrepancy between optimal glottic exposure and failure of tube passage as an important warning sign of occult tracheal pathology and underscores the pivotal role of bronchoscopy and multidisciplinary collaboration in diagnosis and management [5, 8].

Although fewer than 500 cases have been reported worldwide, an increasing number of publications describe TPO presenting for the first time during anesthetic airway management, highlighting its relevance for anesthesiologists despite its rarity [5, 9, 10, 11, 12].

## 2 Methods

This case report was prepared in accordance with the CARE (CAse REport) guidelines. Clinical data were retrospectively collected from the patient's medical records, including perioperative anesthetic management, bronchoscopic findings, histopathological results, and postoperative follow‐up. Written informed consent for publication was obtained from the patient before manuscript submission.

To contextualize the case within the available evidence, we also performed a focused narrative overview of the literature. Relevant reports describing tracheobronchopathia osteochondroplastica presenting as unexpected difficult tracheal intubation were identified through PubMed searches and critically reviewed with respect to clinical presentation, airway management strategies, and perioperative outcomes.

## 3 Case Presentation

A 79‐year‐old woman was scheduled for elective robot‐assisted hysterectomy for gynecological disease. Her medical history included well‐controlled arterial hypertension and type 2 diabetes mellitus treated with oral hypoglycemic agents. She had no previous surgical history and denied respiratory symptoms, including dyspnea, chronic cough, wheezing, or recurrent lower respiratory tract infections.

Preoperative airway assessment was reassuring. She was classified as Mallampati Class II, with an interincisor distance > 3 cm, a thyromental distance > 6 cm, Upper Lip Bite Test (ULBT) Class I, and preserved cervical spine mobility. A preoperative contrast‐enhanced CT examination of the chest and abdomen had been performed as part of the diagnostic work‐up. The examination was not specifically acquired for airway assessment and did not adequately encompass the cervical/subglottic region and proximal trachea subsequently found to be affected. No clinically significant tracheobronchial abnormalities had been reported initially. Following the diagnosis of TPO, retrospective re‐evaluation of the available CT images revealed subtle irregularities of the main bronchi that had not been recognized on the initial interpretation. However, the anatomical coverage of the original examination did not permit adequate retrospective assessment of the affected proximal tracheal segment.

Upon arrival in the operating room, standard American Society of Anesthesiologists monitoring was applied. After adequate preoxygenation, general anesthesia was induced with intravenous propofol, fentanyl, and rocuronium. Neuromuscular blockade was confirmed by train‐of‐four (TOF) monitoring before laryngoscopy. Mask ventilation was immediately effective without the need for adjunctive airway devices.

Videolaryngoscopy provided an excellent laryngeal view (Cormack–Lehane Grade I), with complete visualization of the vocal cords. Despite optimal glottic exposure, a 7.0‐mm internal‐diameter cuffed endotracheal tube (ETT) could not be advanced beyond the glottic plane. A second attempt with a 6.5‐mm ETT was also unsuccessful because of marked resistance immediately below the vocal cords.

Given the discrepancy between the excellent laryngeal view and the inability to advance the ETT, further traumatic intubation attempts were avoided. A 14‐Fr. flexible introducer was advanced through the vocal cords under continuous videolaryngoscopic guidance. The introducer passed smoothly into the trachea and was then used to guide gentle advancement of a 6.0‐mm cuffed ETT, which was successfully positioned without further resistance. Correct tube placement was confirmed by capnography and bilateral chest auscultation. Anesthesia was maintained with sevoflurane, with continuous monitoring of end‐tidal carbon dioxide, invasive arterial pressure, bispectral index, and neuromuscular blockade.

The surgical procedure was completed uneventfully under general anesthesia.

Because the marked resistance encountered during intubation was inconsistent with the reassuring preoperative airway assessment and excellent glottic visualization, an in‐hospital interventional pulmonology consultation was requested after surgery.

Flexible bronchoscopy revealed severe distortion of the upper tracheal lumen with a tight circumferential stenosis that prevented advancement of the bronchoscope beyond the narrowed segment. Given these findings, rigid bronchoscopy was subsequently performed under general anesthesia.

Rigid bronchoscopy allowed passage through the stenotic segment and complete evaluation of the distal trachea. Severe diffuse luminal narrowing was observed, with multiple hard osteocartilaginous nodules arising from prominent tracheal cartilaginous rings. Marked dynamic collapse of the posterior membranous wall was also noted, consistent with associated tracheomalacia.

Mechanical coring‐out of the obstructing osteocartilaginous lesions was performed through rigid bronchoscopy to restore airway patency. Multiple tissue specimens were obtained during the procedure and submitted for histopathological examination.

After bronchoscopic debulking, the endoscopic appearance was considered highly suggestive of tracheobronchopathia osteochondroplastica. Because mechanical recanalization had substantially increased the tracheal lumen diameter, the interventional pulmonologist successfully inserted a 7.0‐mm cuffed endotracheal tube, which was intentionally left in place to maintain airway patency during the immediate postoperative period.

The patient was transferred intubated to the postoperative intensive care unit for continuous airway monitoring and planned protected extubation. Histopathological examination subsequently confirmed tracheobronchopathia osteochondroplastica, demonstrating mature osteocartilaginous tissue beneath the respiratory mucosa.

After a period of intensive monitoring, sedation was gradually discontinued while controlled mechanical ventilation was maintained until respiratory function had fully stabilized. Thirty‐six hours after the bronchoscopic intervention, the patient was successfully extubated without evidence of clinically significant residual airway obstruction or respiratory insufficiency.

The post‐extubation course was uneventful. After an additional 24 h of intensive care observation without respiratory complications, the patient was transferred to the surgical ward. Recovery remained satisfactory, and she was discharged home 72 h later in good clinical condition, without airway‐related complications. A chronological summary of the patient's clinical course is provided in Table 1.

**TABLE 1:** Timeline of the patient's clinical course, highlighting the sequence of perioperative events from preoperative airway assessment to diagnosis of tracheobronchopathia osteochondroplastica, therapeutic rigid bronchoscopy, and successful postoperative recovery.

Time	Clinical event
Preoperative assessment	A 79‐year‐old woman was scheduled for robot‐assisted hysterectomy. Medical history included well‐controlled hypertension and type 2 diabetes mellitus. Airway evaluation was reassuring (Mallampati II, thyromental distance > 6 cm, interincisor distance > 3 cm, ULBT Class I, normal cervical mobility). Preoperative chest CT was not a dedicated airway study and did not adequately encompass the affected proximal tracheal segment
Anesthetic induction	General anesthesia was induced uneventfully. Videolaryngoscopy provided a Cormack–Lehane Grade I view with complete visualization of the vocal cords
Unexpected difficult intubation	Advancement of 7.0‐mm and subsequently 6.5‐mm endotracheal tubes was unsuccessful despite optimal glottic exposure, raising suspicion of subglottic or tracheal obstruction
Airway rescue	A 14‐Fr flexible introducer was successfully advanced beyond the stenotic segment, allowing placement of a 6.0‐mm endotracheal tube and safe completion of surgery
Diagnostic work‐up	Flexible bronchoscopy demonstrated severe proximal tracheal stenosis; rigid bronchoscopy subsequently revealed findings consistent with TPO and associated tracheomalacia
Definitive treatment	The patient underwent rigid bronchoscopy with mechanical coring‐out of the obstructing lesions. Histopathological examination confirmed tracheobronchopathia osteochondroplastica
Postoperative management	A 7.0‐mm endotracheal tube was left in place by the bronchoscopist. The patient was transferred to the intensive care unit for protected extubation
Outcome	Extubation was successfully performed without complications, and the patient recovered uneventfully

### 3.1 Patient Perspective

The patient was informed of the unexpected diagnosis and the need for additional bronchoscopic treatment. She expressed gratitude for the multidisciplinary management that enabled safe completion of the diagnostic and therapeutic pathway and reported satisfaction with the perioperative care received.

## 4 Discussion

Tracheobronchopathia osteochondroplastica (TPO) is an uncommon benign disorder of the central airways characterized by multiple submucosal osteocartilaginous nodules arising from the cartilaginous rings of the trachea and main bronchi, with characteristic sparing of the posterior membranous wall. Its true prevalence remains unknown and is probably underestimated because many patients remain asymptomatic or experience nonspecific respiratory symptoms for years before diagnosis. Consequently, TPO is frequently identified incidentally during bronchoscopy or computed tomography and, less commonly, during airway management for general anesthesia, as in the present case [1, 2, 3, 4].

From an anesthesiologist's perspective, this case illustrates a particularly challenging airway scenario: inability to advance an endotracheal tube despite an excellent laryngoscopic view. Conventional preoperative airway assessment—including Mallampati classification, mouth opening, thyromental distance, Upper Lip Bite Test, and cervical spine mobility—is primarily intended to predict difficult laryngoscopy and mask ventilation rather than distal airway pathology. Accordingly, patients with severe subglottic or tracheal disease may have entirely normal airway predictors and still present with unexpected difficult intubation despite optimal glottic exposure [6].

Current American Society of Anesthesiologists (ASA) Difficult Airway Guidelines emphasize recognition of unexpected resistance during tracheal tube advancement and avoidance of repeated forceful attempts, which may cause airway trauma, bleeding, edema, or complete loss of the airway [6]. Although videolaryngoscopy is highly effective in improving glottic visualization, it cannot identify pathological processes distal to the vocal cords. Therefore, failure of tube advancement despite a Cormack–Lehane Grade I view should raise immediate suspicion of occult subglottic or tracheal abnormalities, including post‐intubation stenosis, tracheal neoplasms, tracheomalacia, inflammatory disorders, and TPO [6, 7].

In our patient, discontinuing repeated attempts with progressively smaller endotracheal tubes and using a flexible introducer proved crucial. This strategy provided atraumatic tracheal access while minimizing mucosal injury and preserving oxygenation until definitive diagnosis could be established. This approach is consistent with current recommendations to limit repeated intubation attempts when unexpected resistance is encountered [6, 7].

Only a limited number of reports describing unexpected difficult intubation secondary to previously undiagnosed TPO have been published in the anesthesiology literature. Warner et al. described a 75‐year‐old man undergoing robot‐assisted prostatectomy in whom repeated attempts with 8.5‐, 8.0‐, and 7.0‐mm endotracheal tubes failed despite a Grade I laryngoscopic view. Subsequent bronchoscopy demonstrated extensive TPO involving the trachea and main bronchi, allowing elective surgery to be postponed until the diagnosis had been established. The authors emphasized that forceful advancement of the endotracheal tube through an unknown obstructive lesion could have resulted in severe bleeding, airway edema, or catastrophic airway loss [9].

Similarly, Erol et al. recently reported a patient in whom TPO was diagnosed only after the inability to advance the endotracheal tube beyond the glottis despite satisfactory laryngoscopic exposure. Bronchoscopy revealed characteristic osteocartilaginous nodules causing subglottic narrowing, again demonstrating that uncomplicated laryngoscopy does not exclude clinically significant distal airway pathology [5].

Takamori et al. reported successful management of a patient with previously diagnosed TPO using bronchoscopically guided tracheal intubation with tube rotation to negotiate the irregular tracheal lumen. Their report showed that careful bronchoscopic guidance may facilitate tube passage by directing the tip through spaces between protruding osteocartilaginous nodules while minimizing airway trauma [13]. Unlike that patient, however, our patient had no previous diagnosis of TPO; preoperative planning was therefore impossible, and airway management required real‐time decision‐making based on unexpected intraoperative findings.

Our case shares the hallmark finding described in previous reports—the discrepancy between excellent glottic visualization and failure of endotracheal tube advancement—but also has several distinctive features. The patient had undergone preoperative contrast‐enhanced CT of the chest and abdomen as part of her diagnostic work‐up; however, this was not a dedicated airway study and did not adequately encompass the cervical/subglottic region and proximal trachea subsequently found to be affected. After the bronchoscopic diagnosis of TPO, retrospective re‐evaluation of the available CT images revealed subtle irregularities of the main bronchi that had not been recognized initially. These findings were not sufficiently conspicuous to raise prospective suspicion of TPO, particularly in an asymptomatic patient with no known tracheal disease and a reassuring airway assessment. Moreover, the limited anatomical coverage precluded adequate retrospective evaluation of the proximal tracheal lesion responsible for difficult tube advancement. This observation illustrates the diagnostic challenge posed by clinically silent TPO and highlights the pivotal role of bronchoscopy when unexplained resistance to endotracheal tube advancement is encountered [2, 3].

Second, airway narrowing was sufficiently severe that successful intubation required placement of a 14‐Fr flexible introducer followed by gentle advancement of a 6.0‐mm endotracheal tube. To our knowledge, this specific rescue strategy has rarely been described in previously unrecognized TPO and may represent a practical airway management option when bronchoscopic equipment is not immediately available or rapid airway control is required while minimizing further airway trauma.

Subsequent bronchoscopy not only established the definitive diagnosis but also enabled immediate therapeutic intervention. Rigid bronchoscopy demonstrated extensive osteocartilaginous nodules associated with severe proximal tracheal stenosis and concomitant tracheomalacia of the posterior membranous wall. Mechanical coring‐out restored an adequate tracheal lumen, allowing placement of a larger 7.0‐mm endotracheal tube and facilitating a controlled postoperative airway strategy. Unlike most previously reported anesthetic cases, in which bronchoscopy served primarily as a diagnostic tool, our patient underwent definitive endoscopic treatment during the same admission, thereby relieving persistent airway obstruction and reducing the risk of subsequent airway compromise [8, 14].

Another noteworthy finding was the coexistence of tracheomalacia. Although TPO classically involves the anterior and lateral tracheal walls while sparing the posterior membranous portion, several reports have described associated dynamic airway collapse, suggesting that chronic airway remodeling and progressive structural changes may extend beyond the cartilaginous lesions themselves [14]. The presence of tracheomalacia further complicated perioperative airway management and strongly influenced the decision to delay extubation until airway stability had been confirmed.

Recent reports further support the association between TPO and unexpected difficult airway management. Wang and Jiang described two patients in whom TPO remained clinically silent until radiological and bronchoscopic investigations revealed extensive tracheobronchial involvement, emphasizing that the disease may remain undetected despite prolonged clinical follow‐up [10]. Yang et al. likewise reported TPO as an unusual cause of failed tracheal intubation during cardiac surgery, reinforcing the need to consider distal airway disorders whenever resistance is encountered below the vocal cords despite uncomplicated laryngoscopy [12]. More recently, Zaki et al. described incidental intraoperative detection of TPO during bronchoscopy in a patient undergoing thyroid surgery, illustrating that the diagnosis may be established during procedures performed for unrelated conditions rather than during evaluation of respiratory symptoms [11]. Collectively, these reports and our experience suggest that anesthesiologists may encounter previously unrecognized TPO more often than traditionally appreciated as videolaryngoscopy and perioperative bronchoscopy become increasingly widespread.

From a therapeutic perspective, most patients with TPO require no specific treatment because the disease generally follows a slowly progressive and often asymptomatic course [1, 2, 3, 4]. Intervention is reserved for patients with clinically significant airway obstruction, recurrent respiratory infections, or disabling symptoms. In these circumstances, rigid bronchoscopy remains a key therapeutic option, allowing mechanical dilation, coring‐out, laser debulking, or removal of obstructing osteocartilaginous lesions while preserving native airway anatomy [8, 14]. In our patient, rigid bronchoscopic coring‐out not only provided tissue for histopathological confirmation but also restored adequate airway patency, avoiding the need for more invasive surgical procedures.

Postoperative airway management represented another critical aspect of this case. Despite successful bronchoscopic recanalization, severe tracheal stenosis, extensive mucosal manipulation, and associated tracheomalacia raised concerns about postoperative airway edema and dynamic airway collapse. The patient therefore remained intubated under close observation in the intensive care unit, and extubation was deliberately delayed for 36 h until airway stability had been confirmed. Although no specific extubation guidelines exist for TPO, this cautious strategy is consistent with principles recommended for complex benign tracheal stenosis and other high‐risk airways [15].

This case also underscores the importance of early multidisciplinary collaboration among anesthesiologists, interventional pulmonologists, thoracic endoscopists, and intensivists. Prompt recognition of abnormal airway findings, timely bronchoscopic assessment, definitive endoscopic treatment, and structured postoperative intensive care were all essential to the favorable outcome. Such coordinated management is particularly valuable in rare airway disorders, in which delayed diagnosis or repeated traumatic airway manipulation may substantially increase perioperative morbidity.

This report supports a practical message for anesthesiologists: failure to advance an endotracheal tube despite excellent glottic visualization should not be attributed solely to technical difficulty. Instead, it should prompt immediate consideration of previously unrecognized subglottic or tracheal pathology, and further forceful intubation attempts should be avoided until the underlying cause has been clarified. Early bronchoscopy may both establish the diagnosis and identify patients requiring immediate therapeutic intervention, as illustrated by this case.

A further consideration is the absence of standardized perioperative algorithms for patients in whom occult tracheal pathology is unexpectedly discovered during airway management. Although the rarity of TPO precludes disease‐specific recommendations, our case suggests that a structured multidisciplinary pathway—including early recognition of failed tube advancement despite optimal laryngoscopy, prompt bronchoscopic evaluation, involvement of interventional pulmonology, consideration of therapeutic rigid bronchoscopy, and planned protected extubation in selected patients—may improve patient safety. Future collaborative efforts should aim to develop practical multidisciplinary algorithms for unexpected subglottic and tracheal obstruction encountered during anesthesia, thereby facilitating decision‐making in these rare but potentially life‐threatening situations.

## 5 Conclusions

Tracheobronchopathia osteochondroplastica should be considered in the differential diagnosis of unexpected difficult tracheal intubation when an endotracheal tube cannot be advanced despite optimal glottic visualization. Conventional preoperative airway assessment may fail to identify clinically significant subglottic or proximal tracheal disease, particularly in asymptomatic patients for whom dedicated upper‐airway imaging is not clinically indicated, making intraoperative recognition essential.

This case highlights several clinically relevant messages. Repeated forceful attempts to advance the endotracheal tube should be avoided when resistance is encountered below the vocal cords because they may cause airway trauma, bleeding, edema, or complete airway loss. Early bronchoscopic evaluation is pivotal not only for establishing the diagnosis but also for identifying patients who may benefit from immediate therapeutic intervention. Successful management of these rare airway disorders also relies on close multidisciplinary collaboration among anesthesiologists, interventional pulmonologists, thoracic endoscopists, and intensivists.

Although tracheobronchopathia osteochondroplastica remains uncommon, increasing reports of incidental diagnosis during anesthetic airway management suggest that anesthesiologists should maintain a high index of suspicion whenever tube advancement is unexpectedly impeded despite uncomplicated laryngoscopy. Greater awareness of this entity may facilitate earlier diagnosis, minimize unnecessary airway trauma, and improve perioperative patient safety.

## Author Contributions

**Ottavio Paolo Volino:** conceptualization, investigation, funding acquisition, writing – original draft, methodology, validation, visualization, writing – review and editing, software, formal analysis, project administration, data curation, supervision, resources. **Loreto Maria:** conceptualization, investigation, validation, methodology, writing – original draft, visualization, writing – review and editing, project administration, formal analysis, software, data curation, supervision, resources. **Palma Antonella:** conceptualization, investigation, validation, methodology, writing – original draft, visualization, writing – review and editing, project administration, formal analysis, software, resources, supervision, data curation. **De Simone Anna:** conceptualization, investigation, validation, data curation, supervision, resources, project administration, formal analysis, software, methodology, visualization, writing – review and editing, writing – original draft. **Tomasello Antonio:** conceptualization, investigation, visualization, writing – original draft, validation, writing – review and editing, methodology, formal analysis, software, project administration, data curation, supervision, resources. **Mainini Marco:** conceptualization, investigation, visualization, data curation, supervision, resources, project administration, formal analysis, software, methodology, validation, writing – review and editing, writing – original draft. **Pisanti Massimo:** conceptualization, investigation, visualization, writing – original draft, methodology, validation, writing – review and editing, project administration, formal analysis, software, resources, supervision, data curation. **Villani Romolo:** conceptualization, investigation, visualization, methodology, validation, writing – original draft, writing – review and editing, project administration, formal analysis, software, data curation, supervision, resources.

## Funding

This work was supported by Department of Woman, Child and General and Specialist Surgery, University of Campania “L. Vanvitelli”, Naples, Italy.

## Ethics Statement

Ethical approval was not required for this case report in accordance with institutional policy and national regulations. The report was prepared in accordance with the ethical principles of the Declaration of Helsinki.

## Consent

Written informed consent for publication of this case report and any accompanying clinical information and images was obtained from the patient. All potentially identifiable personal information was removed to protect patient confidentiality and privacy.

## Conflicts of Interest

The authors declare no conflicts of interest.

## Data Availability

The data supporting the findings of this case report are available from the corresponding author upon reasonable request. No datasets were generated or analyzed beyond the data presented in this article.
