# Correction to “Previously Unrecognized Tracheobronchopathia Osteochondroplastica Diagnosed Following Unexpected Difficult Tracheal Intubation During Robotic Surgery: A Case Report and Literature Overview”

**DOI:** 10.1002/ccr3.73702

**Published:** 2026-10-11

Volino, O. P. , Loreto, M. , Palma, A. , De Simone, A., Tomasello, A., Mainini M., Pisanti, M., Villani, R., “Previously Unrecognized Tracheobronchopathia Osteochondroplastica Diagnosed Following Unexpected Difficult Tracheal Intubation During Robotic Surgery: A Case Report and Literature Overview,” Clinical Case Reports 14, no. 9 (2026): e73504, 10.1002/ccr3.73504 PMC1360176242787569.

The first and last names of the authors, Ottavio Paolo Volino, Maria Loreto, Antonella Palma, Anna De Simone, Antonio Tomasello, Marco Mainini, Massimo Pisanti and Romolo Villani were swapped and have been corrected in the author list and Author Contributions section.

The online version of this article has been corrected accordingly.

We apologize for this error.

